# Plasma lipidomic profiling identifies a novel complex lipid signature associated with ischemic stroke in chronic kidney disease

**Published:** 2020-04-20

**Authors:** Farsad Afshinnia, Adil Jadoon, Thekkelnaycke M. Rajendiran, Tanu Soni, Jaeman Byun, George Michailidis, Subramaniam Pennathur, the Michigan Kidney Translational Core CPROBE Investigator Group

**Affiliations:** 1 University of Michigan, Department of Internal Medicine-Nephrology, Ann Arbor, MI; 2 University of Michigan, Michigan Regional Comprehensive Metabolomics Resource Core, Ann Arbor, MI; 3 University of Michigan, Department of Pathology, Ann Arbor, MI; 4 University of Florida, Department of Statistics, Gainesville, FL; 5 University of Michigan, Department of Molecular and Integrative Physiology, Ann Arbor, MI

**Keywords:** stroke, chronic kidney disease, fatty acids, lipids, mass spectrometry

## Abstract

**Rationale and objective:**

Despite contribution of dyslipidemia to ischemic stroke, plasma lipidomic correlates of stroke in CKD is not studied. This study is aimed to identify plasma lipid alterations associated with stroke.

**Study design:**

Cross sectional.

**Setting and population:**

214 participants of Clinical Phenotyping and Resource Biobank Core (CPROBE). Clinical data and plasma samples at the time of recruitment were obtained and used to generate lipidomic data by liquid chromatography/mass-spectrometry-based untargeted platform.

**Predictors:**

Various levels of free fatty acids, acylcarnitines and complex lipids.

**Outcome:**

Stroke.

**Analytic approach:**

includes compound by compound comparison of lipids using t-test adjusted by false discovery rate in patients with and without stroke, and application of logistic regression analysis to identify independent lipid predictors of stroke and to estimate the odds associated with their various levels.

**Results:**

Overall, we identified 330 compounds. Enrichment analysis revealed overrepresentation of differentially regulated phosphatidylcholines (PC)s and phosphatidylethanolamines (PE)s were overrepresented in stroke (P<0.001). Abundance of PC38:4, PE36:4, PC34:0, and palmitate were significantly higher, but those of plasmenyl-PE (pPE)38:2, and PE 32:2 was significantly lower in patients with stroke (p≤0.0014). After adjusting, each 1-SD increase in palmitate and PC38:4 was independently associated with 1.84 fold (95% CI: 1.06–3.20, p=0.031) and 1.84 fold (1.11–3.05, p=0.018) higher risk of stroke, respectively. We observed a significant trend toward higher abundance of PCs, PEs, pPEs, and sphingomyelins in stroke (p≤0.046).

**Limitations:**

Small sample size; unclear, if similar changes in the same or opposite direction preceded stroke, as the cross-sectional nature of the observation does not allow determining the effect of time course on lipid alterations.

**Conclusion:**

Differential regulation of palmitate, PCs, and PEs in patients with CKD and a history of stroke may represent a previously unrecognized risk factor and might be a target of risk stratification and modification.

## Introduction

Cerebrovascular Accident is a major cause of death and disability in the United States and around the world [1]. Each year over 795,000 people experience stroke in the United States [2] of whom about 140,000 die [3]. Despite a progressive decline in mortality, it has remained the leading cause of long-term disability and costs over 33 billion dollars annually [2]. The Framingham study was pivotal in establishing risk factors for stroke and identified the presence of cardiovascular disease as a major predictor of incident stroke as well as age, systolic blood pressure, use of antihypertensive medications, diabetes mellitus, atrial fibrillation, left ventricular hypertrophy and smoking [4]. More recently, the presence of chronic kidney disease (CKD) defined by an eGFR of <60 ml/min/1.73 m^2^ has emerged as a significant risk factor for stroke [5,6]. Weiner and colleagues used pooled subject-level data from 4 longitudinal community-based studies and demonstrated a higher risk of cardiovascular disease, stroke and death in individuals with clinical CKD [5]. Similarly, Lee and colleagues, also identified CKD as independently associated with incident stroke in a meta-analysis [6]. This increased risk is not completely explained by traditional risk factors, and our current understanding of the role of CKD in the heightened cardiovascular risk remains incomplete. The incidence of CKD has been progressively increasing and the National Center for Health Statistics reports that 15% of the adult US population has some degrees of CKD [7], and are, hence, at a high risk of associated complications.

Several large epidemiological studies of stroke risk have identified dyslipidemia as a risk factor for incident stroke [8,9]. We have shown significant alterations of plasma lipidomics in CKD characterized by increased abundance of palmitate and longer polyunsaturated complex lipids as well as decreased abundance of long chain acylcarnitines by worsening CKD stage [10]. which is explained in part by upregulation of de novo lipogenesis, and mitochondrial β-oxidation of fatty acids [10,11]. Although lipid-lowering agents remain an integral part of all major stoke treatment guidelines [12], the use of statins has only marginally reduced the risk of recurrent stroke [13] and has, in fact, been associated with an increased incidence of hemorrhagic stroke [14], suggesting that there must be mechanisms in place beyond the action of statins on sterol lipids and glycerolipids. Importantly, links with other lipid species such as phospholipids (PLs) and sphingomyelins (SMs) have not been systematically tested. Currently, routine clinical laboratory measurement of lipids is limited to total cholesterol, lipoproteins, and total triglycerides and therefore lacks sufficient coverage of human lipidome. On the other hand, human plasma lipidome consists of thousands of molecular lipid species in over 20 lipid classes [15]. Recent technological advances in mass-spectrometry-based lipidomic platforms have provided the opportunity not only to identify and measure a large array of lipid species in a short period of time, but also to explore their links with phenotypes of interest in a number of clinical settings and understand pathophysiology [10,11,16].

The application of a high-throughput lipidomic platform in patients with CKD may unravel the links between stroke and less-well-studied lipid species. The aim of this study is to identify the differentially regulated lipid species in CKD patients with and without prevalent ischemic stroke. We hypothesize that the plasma abundance and fatty-acyl composition of PLs, SMs, and glycerolipids are different in CKD patients with stroke as compared to CKD patients without stroke. An improved understanding of the alterations in the plasma lipid profile associated with stroke may enhance our ability to identify patients at risk for stroke and target novel mechanistic risk factors.

## Materials and methods

### Patients:

This was a cross-sectional study. The details of patient selection have been published elsewhere [10]. In brief, the study population was patients with CKD from the Clinical Phenotyping Resource and Biobank Core (CPROBE) cohort, a multicenter cohort of patients with CKD established under auspices of the George O’Brien Kidney Center at the University of Michigan, aimed at collecting high-quality data and biosamples for translational research with the Institutional Review Board approval number HUM00020938. We selected 214 patients at various stages of CKD recruited between January of 2009 and July 2012 in an outpatient research setting. All patients were ≥ 18 years of age and were matched by age and sex across all CKD stages. Clinical and laboratory data from the time of recruitment were retrieved. Plasma samples from the time of clinical data gathering were obtained for biomarker identification. We previously showed that this subcohort is unbiased and representative of the entire CPROBE cohort [10]. CKD was defined as an estimated glomerular filtration rate (eGFR) of <60 ml/min/1.73 m^2^, using the CKD Epidemiology Collaboration equation for eGFR calculation. Ischemic stroke was defined as a physician-ascertained, patient-reported prior episodes of central nervous system infarction diagnosed based on clinical evidence, imaging, or other objective evidence [17].

### Biomarker identification:

We used 50 μL of plasma from baseline samples and extracted lipids using the modified Bligh and Dyer method [10,18]. Extracted lipids, dried under nitrogen were resuspended in 100 μL of 10:5:85 acetonitrile:water:isopropyl alcohol followed by 10 mmoL ammonium acetate, and injected to an ABSciex Quadrupole Time of Flight-5600 equipped with a Turbo V ion source (AB Sciex, Concord, Canada) mass spectrometer, using a Shimadzu CTO-20A Nexera 32 UHPLC with Waters Acquity UPLC HSS T3 1.8-mm column (Waters, Milford, MA) with 2 buffers for the mobile phase as detailed elsewhere [10,16]. We identified lipids in both positive and negative modes with a mass range from 50 to 1200 *m/z* and <2 ppm mass error (Supplement Table 1). For quantification, we normalized the peak areas of the extracted ion chromatograms to the peak areas of lipid standards.

### Statistical analysis:

We report mean ± SD or frequency (percentage) for description of baseline variables. For skewed variables, we report median and interquartile range (IQR) and used Kolmogorov-Smirnov test for comparison by two groups. We used t-tests to compare the normally distributed continuous background variables and chi-squared tests for categorical variables. The internal-standard-normalized peak areas of identified lipids were log2 transformed and z-score standardized prior to the downstream analyses. We performed a compound-by-compound analysis to compare the differences in identified lipids in patients with and without stroke using t-tests. To correct for the false discovery rate due to multiple comparisons, we used the Benjamini-Hochberg procedure [19] with Q-value <0.1. Fisher’s exact test was applied to test the enrichment of each lipid class among the top differentially regulated lipids that passed the nominal significance of p<0.05. Mixed linear models were used to illustrate lipid alterations by carbon number and number of double bonds (saturation) within each lipid class in patients with and without stroke. We applied principal component analysis to reduce the number of lipids by aggregating features with high correlation coefficients into aggregate secondary variables within each lipid class using varimax orthogonal transformation [10]. The lipid compositions of the aggregate variables are presented elsewhere [10]. We applied logistic regression models to identify the independent lipids associated with stroke and to estimated odds of stroke associated with alterations of the corresponding lipids. We adjusted the logistic regression models by baseline variables, which were imbalanced in the two groups including age, hypertension, coronary artery disease, peripheral vascular disease, use of statins, and eGFR. The stringent, high-quality control data and reproducibility in this study were described previously [10].

## Results

The mean age of the 214 patients with CKD included in this study was 60 years ± 16 years. The selected cohort consisted of 110 males, 104 females, 150 Caucasians, and 64 African-Americans. Prior to enrollment in the CPROBE cohort and data and plasma collection, 30 patients had a stroke three within the year prior to enrollment, six 1 to 3 years prior to enrollment, five 3 to 5 years prior, fourteen more than 5 years prior. Date of stroke was unknown in 2 patients. The proportion of patients who were African-American, or had hypertension, coronary artery disease, peripheral vascular disease, or used statins was higher in patients with stroke (Table 1). Mean eGFR was also lower in patients with stroke. There were no other significant differences in baseline characteristics (Table 1).

### Compound by compound:

**Figure 1.**
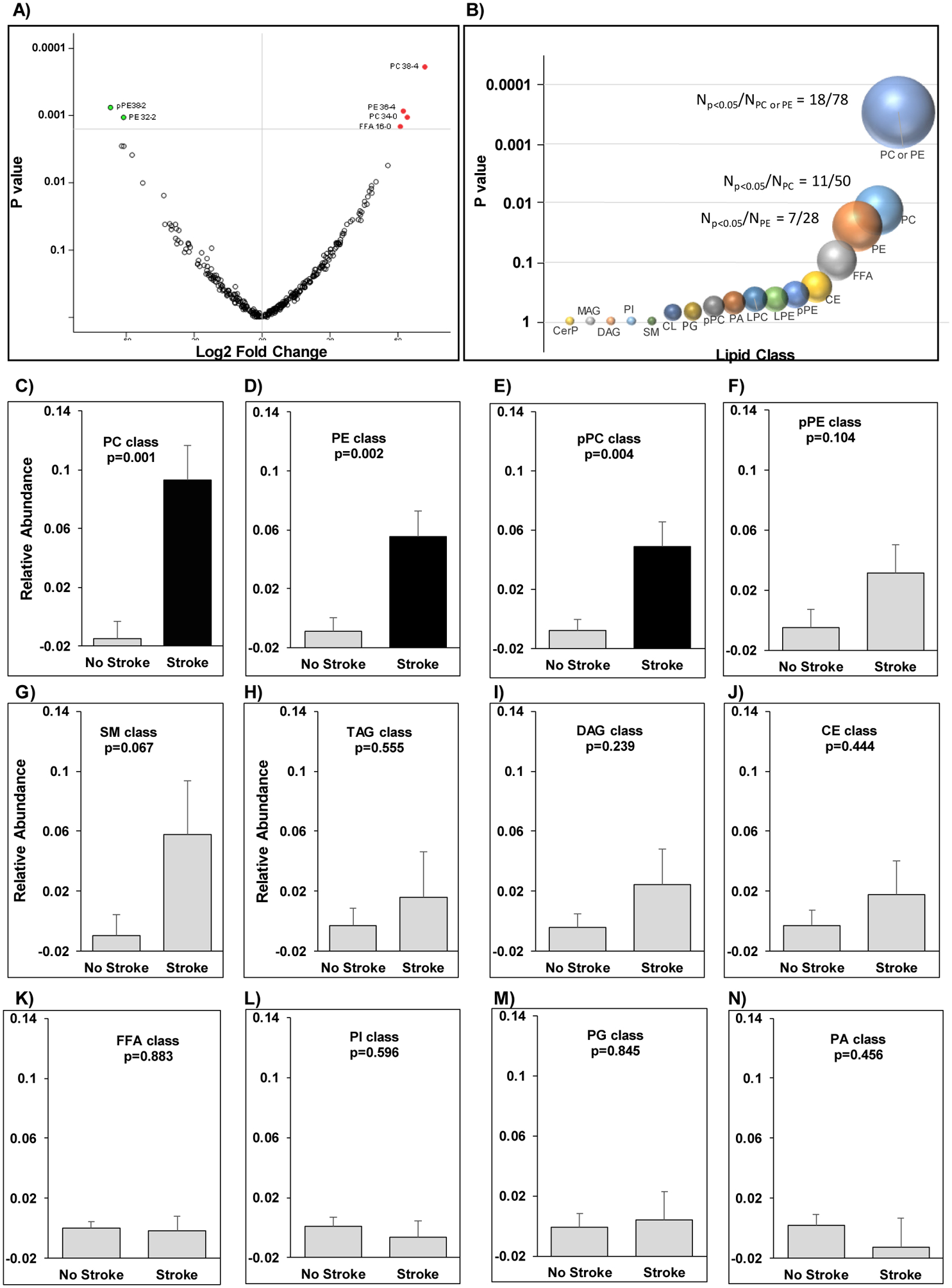
Lipid alterations in patients with and without stroke. (A) Volcano plot demonstrating the fold change of lipids in patient with stroke versus patients without stroke on a log2 scale (X-axis) and their corresponding nominal significance (Y-axis). (B) Enrichment of PCs (p=0.012), PEs (p=0.022), and their combination (Fisher exact p=0.0003). The size of each bubble proportionally increases with statistical significance. (C-N) Mean relative abundance of complex lipids as a class by stroke using t-test. PC: Phosphatidylcholine, PE: Phosphatidylethanolamine; pPE, plasmenyl-PE; FFA: free fatty acid; pPC: plasmenyl-PC, TAG: triacylglycerol, DAG: diacylglycerol, CE: cholesterol esters, SM: sphingomyelin, PI: phosphatidylinositol, PG: phosphatidylglycerol, PA: phosphatidic acid

In our lipidomic analysis of plasma samples, we identified 330 compounds, of which 36 passed the nominal significance threshold (p<0.05) when comparing patients with and without stroke (Supplement Table 2). Only 6 compounds passed the false-discovery-rate threshold (Q<0.1, Figure 1A). Accordingly, the relative abundance of phosphatidylcholine (PC) 38:4, PC 36:4, PC 34:0, and free fatty acid (FFA)16:0 (palmitate) were significantly higher in patients with CKD who had experienced a stoke, and the abundance of plasmenyl-phosphatidylethanolamine (pPE) 38:2, and phosphatidylethanolamine (PE) PE 32:2 were significantly lower (p≤0.0014).

### Class enrichment:

A class enrichment analysis revealed that PCs and PEs, major PLs in biological tissues that perform a variety of essential physiological tasks, were differentially regulated in patients with and without stroke, as evidenced by enrichment of PC and PE lipids with nominally significant p values. Of 36 nominally significant lipids, 11 belonged to the PC class, which consists of 50 lipids (p=0.012), and 7 belonged to the PE class, which consists of 28 lipids (p=0.022). As a single group, PEs and PCs were highly enriched among the top nominally differentiated lipids (p=0.0003, Figure 1B). The overall mean abundance of PCs, PEs, and pPCs as a class in patients with stroke was significantly higher as compared to mean of those classes in patients without stroke (p=0.0001, Figures 1C–1E). No other lipid classes exhibited a difference in relative abundance in patients with CKD with stroke as compared to patients without stroke (Figures 1F–1N).

### Alteration in carbon number and double bonds per molecule:

**Figure 2.**
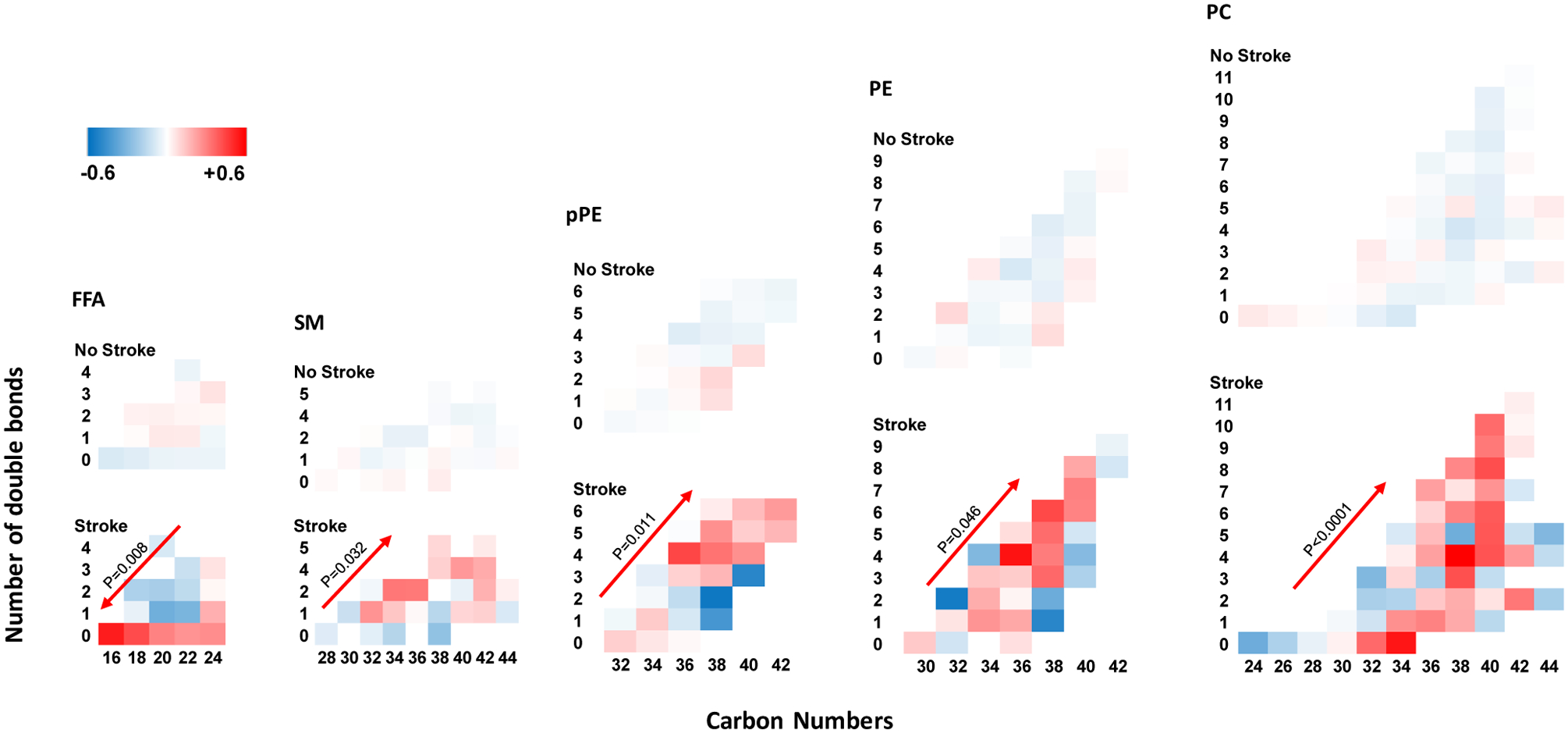
Alteration of lipids by carbon number and number of double bonds. In patients with stroke there was a higher abundance of unsaturated FFAs with lower carbon number. P values are product of an interaction term between carbon number by number of double bonds using a mixed-linear model. FFA: free fatty acid; SM: Sphingomyelin; PE: phosphatidylethanolamine; pPE: plasmenyl-PE; PC: phosphatidylcholine

In patients with stroke, there was a higher abundance of saturated FFAs with lower carbon number (p=0.008, Figure 2). As such, saturated FFAs with lower number of carbons were present in significantly higher abundance as compared with unsaturated longer FFAs. There were no significant alterations in the ratio of FFA carbon number to saturation (number of double bonds) in patients without stroke. Conversely, patients with stroke had a significantly higher abundance of longer complex lipids with a higher number of double bonds in SMs (p=0.032), pPEs (p=0.011), PEs (p=0.046), and PCs (p≤0.001) (Figure 2). There were no significant alterations in the ratio of carbon number to saturation of the complex lipids in patients without stroke.

### Risk estimation:

**Figure 3.**
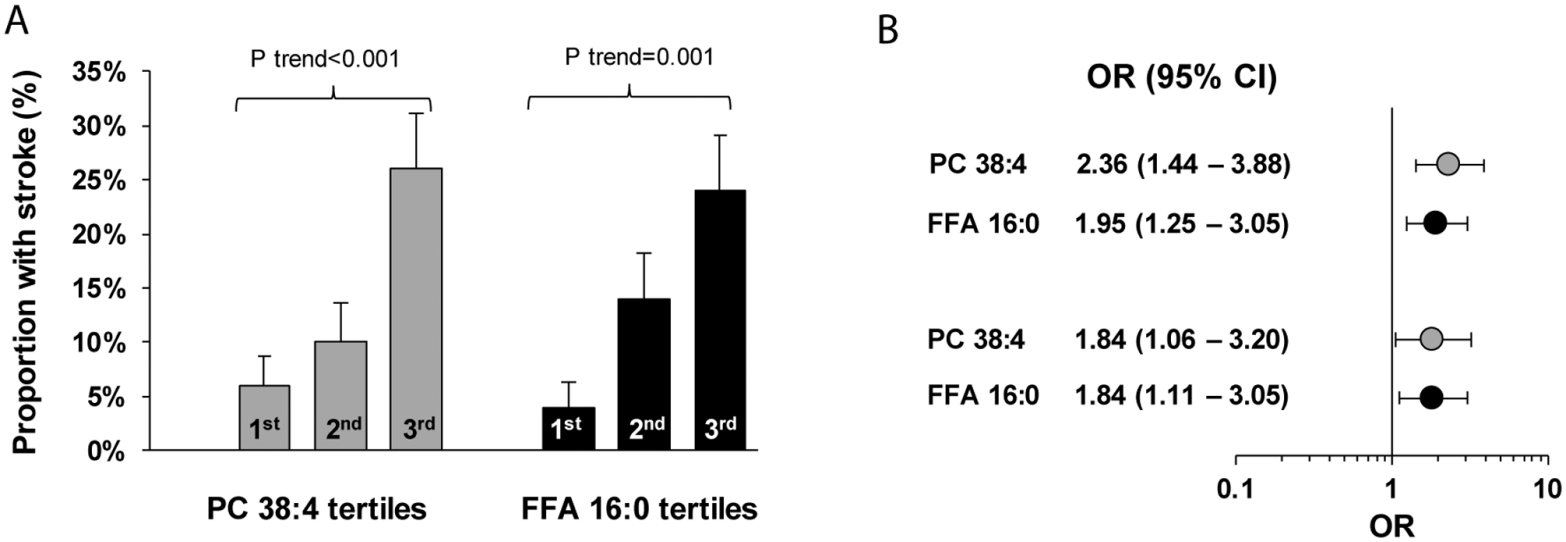
Lipids independently associated with stroke. A. Proportion of patients with stroke in the first (T1), second (T2) and third (T3) tertile of phosphatidylcholine (PC) and unsaturated free fatty acids (FFA)s principal components. B. Odds of stroke by each 1 standard deviation increase in level of PC and unsaturated FFA principal components in unadjusted model as well as after adjusting by age, hypertension, coronary artery disease, peripheral vascular disease, use of statins, and eGFR using multiple logistic regression models. C. Proportion of patients with stroke in the first (T1), second (T2) and third (T3) tertile by PC 38:4 and FFA16:0 abundance. D. Odds of stroke by each 1 standard deviation increase in PC 38:4 and FFA16:0 abundance in unadjusted model as well as after adjusting by age, hypertension, coronary artery disease, peripheral vascular disease, use of statins, and eGFR using multiple logistic regression analysis. PC: phosphatidylcholine; FFA: free fatty acid; OR: odds ratio; CI: confidence interval

To identify independent lipidomic correlates of stroke, we used two approaches including identification of independent lipids at class level and at individual lipids, separately. To identify independent lipid classes with stroke, we utilized logistic regression models with lipid principal components as explanatory variables and identified PC and unsaturated FFAs as independent correlates of stroke. When categorized by their tertiles, we noted an increase in proportion with stroke from 12% in the first tertile to over 53% in the third tertile of PC (linear trend p=0.002), and a decrease from 50% in the first tertile to 10% in the third tertile of unsaturated FFAs as a class (linear trend p=0.004) (Figure 3A). Unadjusted logistic models showed 1.81 fold (95% CI: 1.16 −2.83) higher odds of stroke by each 1-SD increase in PC, and 0.63 fold (95% CI: 0.42 – 0.95) lower odds of stroke by each 1-SD increase in unsaturated FFAs (Figure 2B). After adjusting the associations by age, hypertension, coronary artery disease, peripheral vascular disease, use of statins, and eGFR in the logistic model, there has not been any significant change the associations (Figure 2B).

In the next step we sought the associations of lipids with stroke at individual lipid levels. Using unadjusted multiple logistic regression models, the top 2 lipids that were independently associated with stroke were PC 38:4, and palmitate (FFA16:0). When we categorized the patients into tertiles based on the abundance of these lipids, we noticed a significant linear increase in the proportion of patients with stroke from 6% in the first tertile to 26% in the third tertile for PC 38:4, and from 4% to 24% for palmitate (p≤0.001, Figure 3C). Using an unadjusted logistic model, we noticed 2.36 (95% CI: 1.44 – 3.88) and 1.95 fold (95% CI: 1.25 – 3.05) increase in odds of stroke by each 1-SD increase in abundance of PC38:4 and palmitate, respectively (Figure 3D). After adjusting the model by age, hypertension, coronary artery disease, peripheral vascular disease, use of statins, and eGFR, higher abundance of PC38:4 and palmitate remained significantly associated with higher odds of stroke (Figure 3D).

### Lipid correlates of PC:

**Figure 4.**
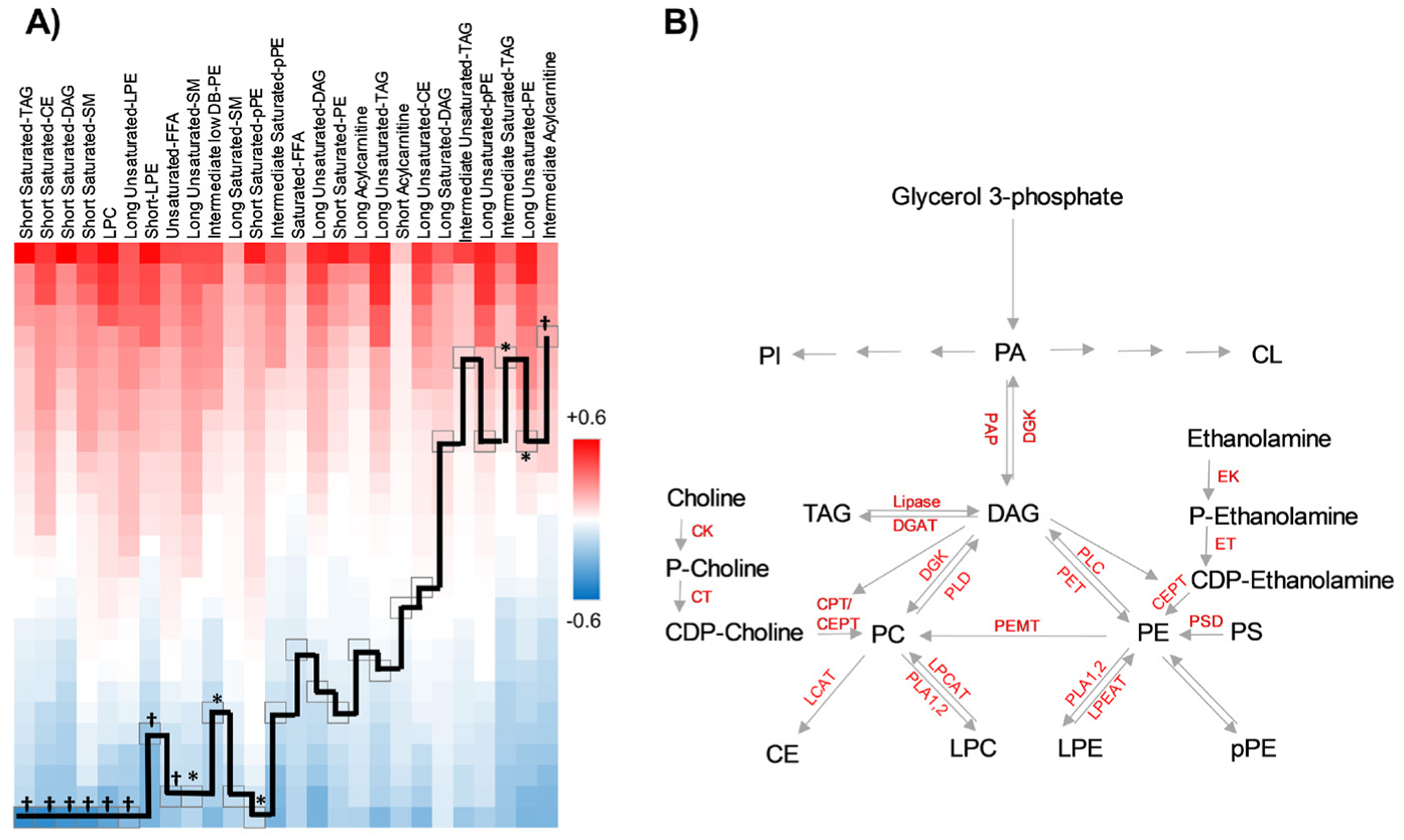
Correlations between PCs and other lipid classes. A. Data-driven illustration of the correlations between PC principal component as a class and other lipids. *p<0.05, †p <0.01. CE: cholesterol esters; DAG: diacylglycerol; PC: phosphatidylcholine; LPC: lyso-PC; PE: phosphatidylethanolamine; LPE: lyso-PE; pPE: plasmenyl-PE; TAG: triacylglycerol; FFA: free fatty acid. B. Known enzymatic pathways leading to generation of PLs and their conversion to other lipids. PA: phosphatidic acid; CL: cardiolipin; PG: phosphatidylglycerol; PI: phosphatidylinositol; PS: phosphatidylserine; CK: choline kinase; CT: CTP:phosphocholine cytidylyltransferase; CEPT: CDP-choline:1,2-diacylglycerol choline/ethanolamine phosphotransferase; CPT: CDP-choline:1,2-diacylglycerol cholinephosphotransferase; DGK: diacylglycerol kinase; DGAT: diacylglycerol acyltransferase; EK: ethanolamine kinase; ET: CTP:phosphoethanolamine cytidylyltransferase; LCAT: lecithin-cholesterol acyltransferase; LPCAT: lysophosphatidylcholine acyltransferase; LPEAT: Lysophospholipid acyltransferase; PAP: phosphatidic acid phosphatase; PET: phosphatidylethanolamine transferase; PEMT: phosphatidylethanolamine N-methyltransferase; PLC: Phospholipase C; PLD: Phospholipase D; PLA1,2: Phospholipase A1,2; PSD: phosphatidylserine decarboxylase

In the next we sought the correlation matrix of PC with other lipid classes ranked from high to low (Figure 4A). We showed that PC as a class was inversely correlated with short saturated TAG, DAG, PE, SM, LPC, short LPE, long unsaturated LPE, SM, unsaturated FFA, and long saturated SM. It was also directly correlated intermediate saturated TAG, long unsaturated PE and intermediate acylcarnitine, and not correlated with the rest of other lipids classes (Figure 4A). The known enzymatic pathways of lipid conversions are shown in Figure 4B.

## Discussion

In this study, we found that PC and PE, as a class, underwent differential regulation in patients with CKD who had experienced a stroke as compared with patients without stroke. Additionally, we noted a significant increase in abundance of PC, PE, pPE, and SM complex lipids with longer carbon chains and more double bonds in patients with stroke. We also observed an increased abundance of palmitate in the plasma of patients who had experienced a stroke. Palmitate, along with PC 38:4, was independently associated with increased odds of stroke in our cohort.

Free fatty acids are the building blocks of complex lipids. They are utilized as precursors to form the esterified complex lipids including PC and PE. In mammalian cells, PC is made by two biosynthetic pathways including CDP-choline pathway and by conversion from PE (Figure 4B) [20]. In the CDP-choline pathway, choline is phosphorylated to phosphocholine by the cytosolic enzyme choline kinase (CK) followed by conversion to CDP-choline by CTP: phosphocholine cytidylyltransferase (CT), an amphitropic protein that is mainly located in the nucleus, and finally, phosphocholine is transferred from CDP-choline to diacylglycerol (DAG) by the integral ER membrane proteins, CDP-choline:1,2-diacylglycerol cholinephosphotransferase (CPT), and to a lesser extent by the dual-specificity protein CDP-choline:1,2-diacylglycerol choline/ethanolamine phosphotransferase (CEPT), resulting in production of PC [20]. In the other PC biosynthetic pathway, PE is converted to PC by three successive methylation reactions catalyzed by phosphatidylethanolamine N-methyltransferase (PEMT) using Sadenosylmethionine as the methyl-group donor [20]. PE is also made by two separate biosynthetic pathways. In the CDP-ethanolamine pathway, similar to the CDP-choline pathway for PC synthesis, ethanolamine is phosphorylated to phosphoethanolamine by the cytosolic enzyme ethanolamine kinase (EK). Another cytosolic protein, CTP: phosphoethanolamine cytidylyltransferase (ET) converts phosphoethanolamine and CTP to CDP-ethanolamine. Finally, CEPT which transfers phosphoethanolamine to DAG to generate PE in the ER. The alternative pathway for PE synthesis, the phosphatidylserine (PS) decarboxylase (PSD) pathway occurs only in mitochondrial inner membranes. PS is imported from its site of synthesis in the ER/MAM to mitochondrial inner membranes where PSD converts PS to PE [20]. Both PC and PE can convert to other lipids via enzymatic reactions (Figure 4B). Subcellular roles of PCs and PEs include regulation of lipoprotein metabolism and VLDL secretion, lipid droplet formation, and control of *de novo* lipogenesis via regulation of sterol regulatory element-binding proteins [20].

The association of differential lipid metabolism with nervous system pathologies has been an area of interest for several decades. Aligned with our observation, in 1975, Ciavatti and colleagues reported a significant increase in plasma palmitic and palmitoleic acid and a significant decrease in linoleic acid in patients with stroke [21]. Similar changes in plasma FFAs associated with stroke have been reported [22]. along with contradictory findings in other studies [22–30]. Such discrepancies in the literature might be explained by the timing of sample collection and dietary fat intake on FFAs and the methods of FFA quantification utilized including enzymatic versus chromatographic assays [31]. Differential regulation of PLs in stroke is reported in a few observations [28,32–34]. In a study comparing 31 patients with lacunar ischemic stroke with 21 healthy controls, several PEs were differentially regulated including [28]. When Liu and colleagues compared 66 patients with ischemic stroke within 9 hours of presentation with 63 control patients, PC (5:0/5:0) was significantly lower in serum of the stroke patients [33]. In a similar case-control study, Sun and colleagues showed that the levels of PC (14:0/20:4) and PC (16:0/22:6) were significantly lower in patients short after ischemic stroke during hospitalization [34]. In our study, PCs and PE’s as lipid class had an overall higher abundance in patients with stroke. At individual lipid species we noted higher abundance of longer polyunsaturated species in stroke, findings which are aligned with previous studies [35,36]. PLs containing polyunsaturated fatty-acyl chains in their sn-2 position increase membrane fluidity and hence my contribute to enhanced insulin sensitivity [37]. All together, these findings suggest that near the time of stroke or prior to that, shorter saturated PLs might be part of an atherogenic lipid profile promoting defective lipoprotein metabolism, altered lipid droplet dynamics, and increased de novo lipogenesis, while increased abundance of longer polyunsaturated PLs long after stroke might be due to upregulation of elongation and desaturation processes of fatty acids as compensatory mechanisms aimed at ameliorating the atherogenic milieu and remodeling of the PLs to less toxic species.

Other putative mechanisms underlying differential regulation of PLs may include alteration in substrate availability for their synthesis, and alterations in the kinetics of the enzymatic pathways involved in their metabolism. FFA substrate availability may, in part, be a reflection of low dietary poly-unsaturated fatty acids in patients with CK [38,39] but may also be a reflection of alteration in *de novo* synthesis of FFA [11]. Decreased lecithin-cholesterol acyltransferase (LCAT) activity in CKD is linked with adverse cardiovascular outcomes [40–42]. Downregulation of phospholipase D is associated with stroke [43], and increased diacylglycerol kinase activity is associated with central nervous system ischemia [44]. Alterations of the activity of any of these enzymes, as well as altered lysophosphatidylcholine acyltransferase (LPCAT) activity [45,46], may contribute to the lipidomic profile associated with stroke in CKD. In our study, the inverse correlates of PC (for example, LPC and short CE, Figure 4A) may reflect blocked conversion of PC to these other lipids, while direct correlates of PC suggests that its high abundance may be explained by upregulation of PEMT mediated conversion of PE to PC or TAG to PC, besides other sources of PC such as CDP-choline mechanism and dietary routes. Other explanations may include altered brain lipid metabolism after brain ischemia, evidenced by alterations captured with MALDI imaging techniques [47–50].

This study has notable strengths. First and foremost, we have applied a high-quality lipidomic platform with excellent reproducibility and rigorous quality control, which minimizes the likelihood of false discovery. Participants were selected from a well-phenotyped cohort of CKD patients with available clinical and laboratory data that allows sufficient adjustment in multivariate models and minimizes the effects of residual confounders. All samples from patients with stage 5 CKD were obtained prior to dialysis or transplant, and therefore, the plasma lipidome was not impacted by renal replacement therapies. This study also has limitations, which include that the observational nature of the study does not allow causal inference, and that it is single-cohort study limiting the generalizability of the findings. Although circumferential evidence such as report of lower levels at the time of stroke besides evidence for upregulation and desaturation of fatty acids which are known as late and compensatory effects long after stroke suggests that higher PC and PE might be a long term compensatory mechanism in our cohort, it remains unclear, if lower PC levels might have promoted stroke in our patients. The sample size is relatively small, and larger studies are required to confirm our findings. We did not have a dietary evaluation of the patients; however similar trends were observed with SM which are less influenced by diet, as were observed for PEs and PCs, so we infer that the role of diet in differential lipid alteration might have been limited.

## Conclusion

In conclusion, high-through put lipidomic analysis identified differential regulation of PCs and PEs in CKD patients with history of stroke compared with patients without stroke. Elevated levels of palmitate and longer polyunsaturated PCs and PEs in stroke offers potential insight into the mechanisms and pathways involved in stroke risk and merits further investigation. The identification of lipid species with higher carbon number and more double bonds among patients with a stroke history is also an intriguing finding and points towards differential metabolism as the etiology of the observed findings. Additional studies are required to study PL changes preceding stroke, which may provide valuable information in prediction and risk stratification strategies beyond traditional risk factors.

## Supplementary Material

Revised Supplementary file

## Figures and Tables

**Table 1.** Baseline characteristics of patients with CKD with and without stroke.

Characteristic^a^	Without Stroke N=184	With Stroke N=30	P value
Age, in years	60±16	60±13	0.824
Male Sex	96 (52.2)	14 (47)	0.576
White race	134 (72.8)	16 (53)	0.031
Current smoker	16 (8.7)	5 (17)	0.174
SBP, in mmHg	135±21	140±24	0.194
DBP, in mmHg	74±11	77±11	0.165
Height, in m	1.7±0.1	1.7±0.1	0.868
Weight, in kg	89±21	97±24	0.057
BMI, in kg/m^2^	30.6±6.5	33.7±8.6	0.065
Comorbidities			
Hypertension	147 (79.9)	29 (97)	0.026
Diabetes	72 (39.1)	17 (57)	0.071
CAD	64 (34.8)	17 (57)	0.022
Heart failure	26 (14.1)	5 (17)	0.779
PVD	20 (10.9)	9 (30)	**0.009**
Medications			
Statins	93 (50.5)	21 (70)	0.048
Fibrates	14 (7.6)	4 (13)	0.291
Niacin	7(3.8)	0 (0)	0.597
Albumin, in g/dL	4.1±0.4	4.0±0.4	0.528
Cholesterol, in mg/dL	172±51	152±54	0.060
LDL-C, in mg/dL	86±40	82±42	0.575
HDL-C, in mg/dL	39±17	35±18	0.273
Triglycerides, in mg/dL	164±112	151±95	0.516
UPCR	1.2 [0.2 to 1.9]	1.2 [0.1 to 1.9]	0.781
WBC, in 1000/μL	7.1±2.8	7.4±3.8	0.590
Creatinine, in mg/dL	2.0±1.1	2.6±1.6	0.052
eGFR, in mL/min	42±23	31±15	0.001
eGFR by categories			
>60 mL/min	35 (19.0)	1 (3)	0.133
30–59 mL/min	85 (46.2)	14 (47)	
15–29 mL/min	50 (27.2)	11 (37)	
< 15 mL/min	14 (7.6)	4 (13)	

a Values are frequency (%) or mean±SD, except for UPCR which is presented as median and interquartile range due to a skewed distribution.

SBP, systolic blood pressure; DBP, diastolic blood pressure; BMI, body mass index; CAD, coronary artery disease; CKD, chronic kidney disease; eGFR, estimated glomerular filtration rate; HDL-C, high density lipoprotein cholesterol; LDL-C, low density lipoprotein cholesterol; PVD, peripheral vascular disease; UPCR, urine protein-creatinine ratio; WBC, white blood cells.
