# Supplementary material for: Plasma lipidomic profiling identifies a novel complex lipid signature associated with ischemic stroke in chronic kidney disease
Source: J Transl Sci. Author manuscript; Available in PMC 2020 Dec 1. (PMC7682927)
Supplement: Revised Supplementary file [file NIHMS1636439-supplement-Revised_Supplementary_file.pdf]

Supplement Table 1: Identified lipids by adducts, mass, and retention time in positive and negative modes.

Lipids in Positive Mode				
**Class**	**Compound name**	**Type of Adducts**	**m/z**	**RT/min**
CE	CE(16:0)	[M+NH4]+	642.6189	10.94
	CE(16:1)	[M+NH4]+	640.6032	10.68
	CE(16:2)	[M+NH4]+	638.5876	10.51
	CE(17:1)	[M+NH4]+	654.6189	10.87
	CE(18:0)	[M+NH4]+	670.6502	11.38
	CE(18:1)	[M+NH4]+	668.6345	10.92
	CE(18:2)	[M+NH4]+	666.6189	10.74
	CE(18:3)	[M+NH4]+	664.6032	10.49
	CE(20:1)	[M+NH4]+	696.6659	11.3
	CE(20:2)	[M+NH4]+	694.6502	11.09
	CE(20:3)	[M+NH4]+	692.6345	10.76
	CE(20:4)	[M+NH4]+	690.6189	10.55
	CE(20:5)	[M+NH4]+	688.6032	10.43
	CE(22:1)	[M+NH4]+	724.6972	11.74
	CE(22:4)	[M+NH4]+	718.6502	10.87
	CE(22:5)	[M+NH4]+	716.6345	10.69
	CE(22:6)	[M+NH4]+	714.6189	10.48
DAG	DAG 28:1	[M+NH4]+	528.4625	6.27
	DAG 30:0	[M+NH4]+	558.5094	7.36
	DAG 30:1	[M+NH4]+	556.4938	6.87
	DAG 32:0	[M+NH4]+	586.5407	7.89
	DAG 32:1	[M+NH4]+	584.5251	7.43
	DAG 32:2	[M+NH4]+	582.5094	7.02
	DAG 32:3	[M+NH4]+	580.4938	6.55
	DAG 33:0	[M+NH4]+	600.5564	8.13
	DAG 33:1	[M+NH4]+	598.5407	7.72
	DAG 34:0	[M+NH4]+	614.572	8.37
	DAG 34:2	[M+NH4]+	610.5407	7.54
	DAG 34:3	[M+NH4]+	608.5251	7.18
	DAG 34:4	[M+NH4]+	606.5094	6.85
	DAG 34:5	[M+NH4]+	604.4938	6.49
	DAG 35:1	[M+NH4]+	626.572	8.21
	DAG 35:2	[M+NH4]+	624.5564	7.83
	DAG 35:3	[M+NH4]+	622.5407	7.45
	DAG 36:0	[M+NH4]+	642.6033	8.85
	DAG 36:1	[M+NH4]+	640.5877	8.42
	DAG 36:2	[M+NH4]+	638.572	8.02

	DAG 36:3	[M+NH4]+	636.5564	7.66
	DAG 36:4	[M+NH4]+	634.5407	7.33
	DAG 36:5	[M+NH4]+	632.5251	6.94
	DAG 36:6	[M+NH4]+	630.5094	6.65
	DAG 38:0	[M+NH4]+	670.6346	8.94
	DAG 38:1	[M+NH4]+	668.6189	8.73
	DAG 38:2	[M+NH4]+	666.6033	8.49
	DAG 38:3	[M+NH4]+	664.5877	8.20
	DAG 38:4	[M+NH4]+	662.572	7.92
	DAG 38:5	[M+NH4]+	660.5564	7.57
	DAG 38:6	[M+NH4]+	658.5407	7.27
	DAG 38:7	[M+NH4]+	656.5251	6.88
	DAG 40:0	[M+NH4]+	698.6659	9.56
	DAG 40:5	[M+NH4]+	688.5877	8.03
	DAG 40:6	[M+NH4]+	686.572	7.74
	DAG 40:7	[M+NH4]+	684.5564	7.51
	DAG 40:8	[M+NH4]+	682.5407	7.13
LPC	LPC 14:0	[M+H]+	468.309	1.08
	LPC 14:0	[M+Na]+	490.291	1.05
	LPC 15:0	[M+H]+	482.3246	1.20
	LPC 15:0	[M+Na]+	504.3066	1.21
	LPC 16:0	[M+H]+	496.3403	1.46
	LPC 16:0	[M+Na]+	504.343	1.82
	LPC 16:1	[M+Na]+	516.3066	1.14
	LPC 17:0	[M+Na]+	532.3379	2.20
	LPC 17:1	[M+H]+	508.3403	1.40
	LPC 18:0	[M+H]+	524.3716	2.30
	LPC 18:0	[M+Na]+	530.3586	2.03
	LPC 18:1	[M+Na]+	544.3379	1.81
	LPC 18:2	[M+Na]+	542.3223	1.09
	LPC 18:3	[M+H]+	518.3246	1.09
	LPC 18:3	[M+Na]+	540.3066	1.05
	LPC 19:0	[M+H]+	538.3873	2.73
	LPC 19:0	[M+Na]+	560.3692	2.76
	LPC 20:0	[M+H]+	552.4029	3.22
	LPC 20:0	[M+Na]+	574.3848	3.17
	LPC 20:1	[M+H]+	550.3873	2.48
	LPC 20:1	[M+Na]+	572.3692	2.47
	LPC 20:2	[M+H]+	548.3716	1.86
	LPC 20:3	[M+Na]+	568.3379	1.42
	LPC 20:5	[M+H]+	542.3246	1.01

	LPC 22:0	[M+H]+	580.4342	4.13
	LPC 22:4	[M+H]+	572.3716	1.76
	LPC 22:4	[M+Na]+	594.3535	1.82
	LPC 22:5	[M+H]+	570.356	1.47
	LPC 22:5	[M+Na]+	592.3379	1.45
	LPC 24:0	[M+H]+	608.4655	5.03
	LPC 24:1	[M+H]+	606.4498	4.18
	LPC 26:1	[M+H]+	634.4812	5.02
LPE	LPE 16:0	[M+H]+	454.2934	1.62
	LPE 18:0	[M+H]+	482.3246	2.45
	LPE 18:0	[M+Na]+	504.3066	2.46
	LPE 18:1	[M+H]+	480.309	1.76
	LPE 18:2	[M+H]+	478.2934	1.32
	LPE 18:3	[M+H]+	476.2777	1.08
	LPE 20:3	[M+H]+	504.309	1.55
	LPE 20:4	[M+H]+	502.2934	1.25
	LPE 22:5	[M+H]+	528.309	1.46
	LPE 22:6	[M+H]+	526.2933	1.19
MAG	MAG 17:0	[M+Li]+	351.3087	3.12
	MAG 18:1	[M+NH4]+	374.327	2.90
	MAG 18:2	[M+NH4]+	372.3114	2.26
PA	PA 34:0	[M+Na]+	699.4941	13.75
PC	PC 28:0	[M+Na]+	700.4893	5.11
	PC 30:0	[M+Na]+	728.5206	5.85
	PC 30:1	[M+Na]+	726.505	5.33
	PC 32:0	[M+H]+	734.57	5.36
	PC 32:1	[M+Na]+	754.5363	5.99
	PC 32:2	[M+Na]+	752.5206	5.51
	PC 33:3	[M+H]+	742.5387	5.39
	PC 34:0	[M+Na]+	784.5832	7.07
	PC 34:3	[M+Na]+	778.5363	5.65
	PC 34:4	[M+H]+	754.5387	6.02
	PC 34:4	[M+Na]+	776.5206	5.49
	PC 35:5	[M+H]+	766.5387	5.41
	PC 36:1	[M+Na]+	810.5989	7.18
	PC 36:3	[M+H]+	784.5856	7.10
	PC 36:4	[M+H]+	782.57	4.68
	PC 36:4	[M+Na]+	804.5519	5.85
	PC 36:5	[M+H]+	780.5543	4.28
	PC 36:5	[M+Na]+	802.5363	5.58
	PC 36:6	[M+Na]+	800.5206	5.31

	PC 36:7	[M+H]+	776.523	5.25
	PC 38:4	[M+Na]+	832.5832	6.75
	PC 38:5	[M+H]+	808.5856	5.06
	PC 38:6	[M+H]+	806.57	4.85
	PC 38:6	[M+Na]+	828.5519	5.99
	PC 38:7	[M+H]+	804.5543	5.87
	PC 38:7	[M+Na]+	826.5363	5.46
	PC 38:8	[M+H]+	802.5387	5.41
	PC 40:10	[M+H]+	826.5387	5.55
	PC 40:8	[M+Na]+	852.5519	5.70
	PC 40:9	[M+H]+	828.5543	5.86
	PC 42:10	[M+H]+	854.57	5.63
	PC 42:11	[M+H]+	852.5543	5.68
	PC 42:9	[M+H]+	856.5856	6.69
PE	PE 34:1	[M+H]+	718.5387	6.74
	PE 34:2	[M+H]+	716.523	6.29
	PE 34:3	[M+H]+	714.5074	5.94
	PE 35:1	[M+H]+	732.5543	6.99
	PE 35:2	[M+H]+	730.5387	6.66
	PE 36:0	[M+H]+	748.5856	7.76
	PE 36:1	[M+H]+	746.57	7.35
	PE 36:2	[M+H]+	744.5543	6.89
	PE 36:4	[M+H]+	740.523	6.31
	PE 36:5	[M+H]+	738.5074	5.88
	PE 37:6	[M+H]+	750.5074	5.86
	PE 38:3	[M+H]+	770.57	7.15
	PE 38:4	[M+H]+	768.5543	6.90
	PE 38:6	[M+H]+	764.523	6.10
	PE 40:6	[M+H]+	792.5543	6.86
	PE 40:7	[M+H]+	790.5387	6.27
pPC	pPC 18:0	[M+Na]+	544.3379	1.73
	pPC 20:0	[M+Na]+	572.3692	2.45
	pPC 36:1	[M+Na]+	794.604	7.48
	pPC 36:4	[M+Na]+	788.557	6.45
	pPC 40:4	[M+Na]+	844.6196	7.40
	pPC 44:4	[M+Na]+	900.6822	8.16
pPE	pPE 34:0	[M+H]+	704.5594	7.55
	pPE 34:1	[M+H]+	702.5438	7.10
	pPE 34:2	[M+H]+	700.5281	6.65
	pPE 36:1	[M+H]+	730.5751	7.62
	pPE 36:2	[M+H]+	728.5594	7.20

	pPE 36:4	[M+H]+	724.5281	6.58
	pPE 36:5	[M+H]+	722.5125	6.25
	pPE 38:1	[M+H]+	758.6063	8.08
	pPE 38:2	[M+H]+	756.5907	7.81
	pPE 38:3	[M+H]+	754.5751	7.42
	pPE 38:4	[M+H]+	752.5594	7.21
	pPE 38:5	[M+H]+	750.5438	6.71
	pPE 38:6	[M+H]+	748.5281	6.49
	pPE 40:4	[M+H]+	780.5907	7.70
	pPE 40:6	[M+H]+	776.5594	7.12
SM	SM 21:1	[M]+	521.3719	1.26
	SM 28:0	[M]+	621.4971	3.83
	SM 30:1	[M+Na]+	669.4948	4.26
	SM 31:1	[M]+	661.5284	4.66
	SM 32:0	[M+Na]+	699.5417	5.36
	SM 32:1	[M+Na]+	697.5261	4.99
	SM 32:2	[M]+	673.5284	4.50
	SM 32:2	[M+Na]+	695.5104	4.43
	SM 33:1	[M+Na]+	711.5417	5.37
	SM 33:2	[M]+	687.5441	4.85
	SM 33:2	[M+Na]+	709.5261	4.82
	SM 34:0	[M+Na]+	727.573	6.03
	SM 34:1	[M]+	703.5754	5.73
	SM 34:1	[M+Na]+	725.5573	5.72
	SM 34:2	[M+Na]+	723.5417	5.17
	SM 35:0	[M]+	719.6067	6.56
	SM 35:1	[M+Na]+	739.573	6.07
	SM 35:2	[M+Na]+	737.5573	5.59
	SM 36:1	[M+Na]+	753.5886	6.46
	SM 36:2	[M]+	729.5911	5.89
	SM 36:2	[M+Na]+	751.573	5.94
	SM 37:1	[M]+	745.6224	6.81
	SM 37:2	[M]+	743.6067	6.29
	SM 38:0	[M]+	761.6536	7.33
	SM 38:1	[M+Na]+	781.6199	7.07
	SM 38:4	[M]+	753.5911	5.57
	SM 38:5	[M]+	751.5754	5.12
	SM 39:1	[M+Na]+	795.6356	7.40
	SM 39:2	[M]+	771.638	6.84
	SM 40:1	[M+Na]+	809.6513	7.59
	SM 40:2	[M]+	785.6536	6.88

	SM 40:2	[M+Na]+	807.6356	7.18
	SM 40:4	[M]+	781.6224	6.24
	SM 41:0	[M]+	803.7006	8.09
	SM 41:1	[M+Na]+	823.6669	7.87
	SM 41:2	[M+Na]+	821.6513	7.42
	SM 41:4	[M]+	795.638	6.50
	SM 42:1	[M+Na]+	837.6826	8.12
	SM 42:2	[M]+	813.6849	7.64
	SM 42:2	[M+Na]+	835.6669	7.64
	SM 42:4	[M]+	809.6536	6.81
	SM 42:5	[M]+	807.638	6.45
	SM 43:1	[M]+	829.7163	8.43
	SM 43:1	[M+Na]+	851.6982	8.31
	SM 43:2	[M]+	827.7006	8.05
	SM 43:2	[M+Na]+	849.6826	7.79
	SM 43:4	[M]+	823.6693	7.01
	SM 44:1	[M]+	843.7319	8.60
	SM 44:2	[M]+	841.7163	8.13
TAG	TAG 40:0	[M+NH4]+	712.6451	9.49
	TAG 42:0	[M+Na]+	745.6318	9.77
	TAG 42:0	[M+NH4]+	740.6764	9.76
	TAG 42:1	[M+NH4]+	738.6608	9.50
	TAG 44:1	[M+NH4]+	766.6921	9.82
	TAG 44:2	[M+NH4]+	764.6764	9.53
	TAG 46:0	[M+NH4]+	796.739	10.31
	TAG 46:1	[M+NH4]+	794.7233	10.07
	TAG 46:2	[M+NH4]+	792.7077	9.83
	TAG 46:3	[M+NH4]+	790.6921	9.59
	TAG 48:0	[M+NH4]+	824.7702	10.54
	TAG 48:1	[M+Na]+	827.71	8.98
	TAG 48:1	[M+NH4]+	822.7546	10.31
	TAG 48:2	[M+NH4]+	820.739	10.09
	TAG 48:3	[M+NH4]+	818.7233	9.86
	TAG 49:0	[M+NH4]+	838.7859	10.68
	TAG 49:1	[M+NH4]+	836.7702	10.48
	TAG 49:2	[M+NH4]+	834.7546	10.24
	TAG 50:0	[M+NH4]+	852.8015	10.80
	TAG 50:1	[M+Na]+	855.7413	9.29
	TAG 50:1	[M+NH4]+	850.7859	10.54
	TAG 50:2	[M+NH4]+	848.7702	10.34
	TAG 50:3	[M+NH4]+	846.7546	10.13

	TAG 50:4	[M+NH4]+	844.739	9.93
	TAG 50:5	[M+NH4]+	842.7233	9.74
	TAG 51:1	[M+NH4]+	864.8015	10.71
	TAG 51:2	[M+NH4]+	862.7859	10.51
	TAG 51:3	[M+NH4]+	860.7702	10.31
	TAG 51:4	[M+NH4]+	858.7546	10.11
	TAG 52:0	[M+NH4]+	880.8328	11.04
	TAG 52:1	[M+Na]+	883.7726	10.68
	TAG 52:1	[M+NH4]+	878.8172	10.82
	TAG 52:2	[M+NH4]+	876.8015	10.57
	TAG 52:3	[M+Na]+	879.7413	10.40
	TAG 52:3	[M+NH4]+	874.7859	10.38
	TAG 52:4	[M+Na]+	877.7257	10.22
	TAG 52:4	[M+NH4]+	872.7702	10.19
	TAG 52:5	[M+Na]+	875.71	10.01
	TAG 52:5	[M+NH4]+	870.7546	9.99
	TAG 52:6	[M+Na]+	873.6944	9.80
	TAG 52:6	[M+NH4]+	868.739	9.81
	TAG 52:7	[M+NH4]+	866.7233	9.61
	TAG 53:0	[M+NH4]+	894.8484	11.16
	TAG 53:1	[M+NH4]+	892.8328	10.93
	TAG 53:2	[M+NH4]+	890.8172	10.74
	TAG 53:3	[M+NH4]+	888.8015	10.54
	TAG 53:4	[M+NH4]+	886.7859	10.35
	TAG 53:5	[M+NH4]+	884.7702	10.17
	TAG 53:6	[M+NH4]+	882.7546	9.96
	TAG 53:7	[M+NH4]+	880.739	9.77
	TAG 54:0	[M+Na]+	913.8195	11.28
	TAG 54:0	[M+NH4]+	908.8641	11.28
	TAG 54:1	[M+Na]+	911.8038	11.03
	TAG 54:1	[M+NH4]+	906.8484	11.06
	TAG 54:2	[M+Na]+	909.7882	10.87
	TAG 54:2	[M+NH4]+	904.8328	10.85
	TAG 54:3	[M+Na]+	907.7726	10.69
	TAG 54:3	[M+NH4]+	902.8172	10.66
	TAG 54:4	[M+NH4]+	900.8015	10.44
	TAG 54:5	[M+Na]+	903.7413	10.33
	TAG 54:5	[M+NH4]+	898.7859	10.24
	TAG 54:6	[M+Na]+	901.7257	10.06
	TAG 54:6	[M+NH4]+	896.7702	10.06
	TAG 54:7	[M+Na]+	899.71	9.85

	TAG 54:7	[M+NH4]+	894.7546	9.90
	TAG 54:8	[M+NH4]+	892.739	9.71
	TAG 55:0	[M+NH4]+	922.8797	11.39
	TAG 55:1	[M+NH4]+	920.8641	11.17
	TAG 55:3	[M+NH4]+	916.8328	10.77
	TAG 55:5	[M+NH4]+	912.8015	10.46
	TAG 55:6	[M+NH4]+	910.7859	10.29
	TAG 56:0	[M+NH4]+	936.8954	11.54
	TAG 56:1	[M+NH4]+	934.8797	11.30
	TAG 56:2	[M+NH4]+	932.8641	11.09
	TAG 56:3	[M+NH4]+	930.8484	10.88
	TAG 56:4	[M+NH4]+	928.8328	10.71
	TAG 56:5	[M+Na]+	931.7726	10.57
	TAG 56:5	[M+NH4]+	926.8172	10.55
	TAG 56:6	[M+NH4]+	924.8015	10.38
	TAG 56:7	[M+Na]+	927.7413	10.20
	TAG 56:7	[M+NH4]+	922.7859	10.20
	TAG 56:8	[M+NH4]+	920.7702	10.02
	TAG 56:9	[M+Na]+	923.71	9.87
	TAG 56:9	[M+NH4]+	918.7546	9.82
	TAG 57:2	[M+NH4]+	946.8797	11.23
	TAG 57:3	[M+NH4]+	944.8641	11.00
	TAG 57:6	[M+NH4]+	938.8172	10.50
	TAG 57:7	[M+NH4]+	936.8015	10.36
	TAG 57:8	[M+NH4]+	934.7859	10.17
	TAG 58:0	[M+NH4]+	964.9267	11.85
	TAG 58:1	[M+NH4]+	962.911	11.57
	TAG 58:10	[M+NH4]+	944.7702	9.95
	TAG 58:11	[M+NH4]+	942.7546	9.77
	TAG 58:12	[M+NH4]+	940.739	9.57
	TAG 58:2	[M+NH4]+	960.8954	11.32
	TAG 58:3	[M+NH4]+	958.8797	11.12
	TAG 58:4	[M+NH4]+	956.8641	10.93
	TAG 58:5	[M+NH4]+	954.8484	10.80
	TAG 58:6	[M+NH4]+	952.8328	10.60
	TAG 58:7	[M+NH4]+	950.8172	10.50
	TAG 58:8	[M+NH4]+	948.8015	10.29
	TAG 58:9	[M+NH4]+	946.7859	10.14
	TAG 60:10	[M+NH4]+	972.8015	10.18
	TAG 60:11	[M+NH4]+	970.7859	10.09
	TAG 60:12	[M+NH4]+	968.7702	9.90

	TAG 60:15	[M+NH4]+	962.7233	9.31
	TAG 60:3	[M+NH4]+	986.911	11.24
	TAG 60:8	[M+NH4]+	976.8328	10.53
	TAG 62:12	[M+NH4]+	996.8015	10.17
	TAG 62:14	[M+NH4]+	992.7702	9.84
	TAG 62:3	[M+NH4]+	1014.942	11.58

**Lipids in Negative Mode**				
**Class**	**Sample.Name**	**Adducts**	**m/z**	**RT/min**
FFA	FFA (16:0)		255.2329	2.30
	FFA(18:0)		283.2642	3.27
	FFA(18:1)		281.2486	2.50
	FFA(18:2)		279.2329	1.92
	FFA(20:0)		311.2955	4.30
	FFA(20:1)		309.2799	3.45
	FFA(20:2)		307.2642	2.82
	FFA (20:4)		303.2329	1.85
	FFA(22:0)		339.3268	5.29
	FFA(22:1)		337.3112	4.43
	FFA(22:2)		335.2955	3.76
	FFA(22:3)		333.2799	3.18
	FFA(24:0)		367.3581	6.21
	FFA(24:1)		365.3425	5.36
	FFA(24:2)		363.3268	4.70
	FFA(24:3)		361.3112	4.07
CerP	CerP 32:1	[M-H]-	588.4393	5.02
	CerP 34:1	[M-H]-	616.4706	5.75
CL	CL 66:1	[M-2H](2-)	688.4861	6.17
	CL 70:5	[M-2H](2-)	712.4861	5.95
	CL 72:3	[M-2H](2-)	728.5174	7.52
	CL 74:1	[M-2H](2-)	744.5487	6.75
	CL 74:3	[M-2H](2-)	742.5331	5.67
	CL 74:5	[M-2H](2-)	740.5174	5.83
	CL 74:7	[M-2H](2-)	738.5018	6.03
	CL 76:11	[M-2H](2-)	748.4861	5.88
	CL 78:3	[M-2H](2-)	770.5644	7.41
	CL 78:5	[M-2H](2-)	768.5487	6.31
	CL 82:13	[M-2H](2-)	788.5174	6.32
	CL 82:9	[M-2H](2-)	792.5487	7.01
LPE	LPE 16:0	[M-H]-	452.2777	1.60
	LPE 17:0	[M-H]-	466.2934	1.92

	LPE 18:0	[M-H]-	480.309	2.34
	LPE 18:1	[M-H]-	478.2934	1.76
	LPE 18:2	[M-H]-	476.2777	1.31
	LPE 20:3	[M-H]-	502.2934	1.59
	LPE 20:4	[M-H]-	500.2777	1.29
	LPE 20:5	[M-H]-	498.2621	1.05
	LPE 22:4	[M-H]-	528.309	1.85
	LPE 22:5	[M-H]-	526.2934	1.49
	LPE 22:6	[M-H]-	524.2777	1.26
	LPE 24:0	[M-H]-	564.403	5.18
PA	PA 34:1	[M-H]-	673.4808	6.58
	PA 34:2	[M-H]-	671.4652	6.15
	PA 36:4	[M-H]-	695.4652	6.08
	PA 38:6	[M-H]-	719.4652	6.03
PC	PC 24:0	[M-Ac-H]-	680.4503	3.58
	PC 26:0	[M-Ac-H]-	708.4816	4.38
	PC 28:0	[M-Ac-H]-	736.5129	5.15
	PC 29:0	[M-Ac-H]-	750.5285	5.48
	PC 30:0	[M-Ac-H]-	764.5442	5.87
	PC 30:1	[M-Ac-H]-	762.5285	5.31
	PC 31:0	[M-Ac-H]-	778.5598	6.03
	PC 32:0	[M-Ac-H]-	792.5754	6.50
	PC 32:1	[M-Ac-H]-	790.5598	6.01
	PC 32:2	[M-Ac-H]-	788.5442	5.51
	PC 32:3	[M-Ac-H]-	786.5285	5.06
	PC 33:0	[M-Ac-H]-	806.5911	6.71
	PC 33:1	[M-Ac-H]-	804.5754	6.33
	PC 33:2	[M-Ac-H]-	802.5598	5.87
	PC 33:3	[M-Ac-H]-	800.5442	5.43
	PC 34:0	[M-Ac-H]-	820.6068	7.06
	PC 34:1	[M-Ac-H]-	818.5911	6.56
	PC 34:2	[M-Ac-H]-	816.5754	4.60
	PC 34:3	[M-Ac-H]-	814.5598	5.69
	PC 34:4	[M-Ac-H]-	812.5442	5.46
	PC 34:5	[M-Ac-H]-	810.5285	5.05
	PC 35:1	[M-Ac-H]-	832.6068	6.91
	PC 35:2	[M-Ac-H]-	830.5911	6.45
	PC 35:3	[M-Ac-H]-	828.5754	6.02
	PC 35:4	[M-Ac-H]-	826.5598	5.85
	PC 35:5	[M-Ac-H]-	824.5442	5.42
	PC 36:1	[M-Ac-H]-	846.6224	7.16

	PC 36:2	[M-Ac-H]-	844.6068	6.72
	PC 36:3	[M-Ac-H]-	842.5911	4.71
	PC 36:4	[M-Ac-H]-	840.5754	4.96
	PC 36:5	[M-Ac-H]-	838.5598	5.57
	PC 36:6	[M-Ac-H]-	836.5442	5.34
	PC 37:1	[M-Ac-H]-	860.6381	7.39
	PC 37:2	[M-Ac-H]-	858.6224	7.06
	PC 37:3	[M-Ac-H]-	856.6068	6.65
	PC 37:4	[M-Ac-H]-	854.5911	6.44
	PC 37:5	[M-Ac-H]-	852.5754	5.96
	PC 37:6	[M-Ac-H]-	850.5598	5.71
	PC 38:1	[M-Ac-H]-	874.6537	7.6
	PC 38:2	[M-Ac-H]-	872.6381	7.32
	PC 38:3	[M-Ac-H]-	870.6224	6.97
	PC 38:4	[M-Ac-H]-	868.6068	6.65
	PC 38:5	[M-Ac-H]-	866.5911	6.27
	PC 38:6	[M-Ac-H]-	864.5754	5.92
	PC 38:7	[M-Ac-H]-	862.5598	5.45
	PC 39:4	[M-Ac-H]-	882.6224	6.98
	PC 39:5	[M-Ac-H]-	880.6068	6.53
	PC 39:6	[M-Ac-H]-	878.5911	6.32
	PC 40:1	[M-Ac-H]-	902.685	8.19
	PC 40:2	[M-Ac-H]-	900.6694	7.94
	PC 40:3	[M-Ac-H]-	898.6537	7.42
	PC 40:4	[M-Ac-H]-	896.6381	7.19
	PC 40:5	[M-Ac-H]-	894.6224	6.94
	PC 40:6	[M-Ac-H]-	892.6068	6.58
	PC 40:7	[M-Ac-H]-	890.5911	6.10
	PC 40:8	[M-Ac-H]-	888.5754	5.70
	PC 40:9	[M-Ac-H]-	886.5598	5.30
	PC 42:10	[M-Ac-H]-	912.5754	5.63
	PC 42:2	[M-Ac-H]-	928.7007	8.38
	PC 42:4	[M-Ac-H]-	924.6694	7.84
	PC 42:5	[M-Ac-H]-	922.6537	7.32
	PC 42:7	[M-Ac-H]-	918.6224	6.73
	PC 44:2	[M-Ac-H]-	956.7319	8.86
	PC 44:4	[M-Ac-H]-	952.7007	8.37
	PC 44:5	[M-Ac-H]-	950.685	7.86
PE	PE 30:0	[M-H]-	662.4761	6.03
	PE 32:0	[M-H]-	690.5074	6.65
	PE 32:1	[M-H]-	688.4917	6.18

	PE 32:2	[M-H]-	686.4761	5.65
	PE 33:1	[M-H]-	702.5074	6.47
	PE 33:2	[M-H]-	700.4917	6.02
	PE 34:1	[M-H]-	716.5231	6.78
	PE 34:2	[M-H]-	714.5074	5.02
	PE 34:2	[M-H]-	714.5074	6.31
	PE 34:3	[M-H]-	712.4917	5.98
	PE 34:4	[M-H]-	710.4761	5.64
	PE 35:0	[M-H]-	732.5543	7.49
	PE 35:1	[M-H]-	730.5387	7.05
	PE 35:2	[M-H]-	728.5231	6.60
	PE 35:3	[M-H]-	726.5074	6.17
	PE 35:4	[M-H]-	724.4917	6.00
	PE 36:0	[M-H]-	746.57	7.77
	PE 36:1	[M-H]-	744.5543	7.35
	PE 36:2	[M-H]-	742.5387	6.91
	PE 36:3	[M-H]-	740.5231	6.46
	PE 36:4	[M-H]-	738.5074	6.14
	PE 36:5	[M-H]-	736.4917	5.88
	PE 37:2	[M-H]-	756.5543	6.15
	PE 37:3	[M-H]-	754.5387	6.74
	PE 37:4	[M-H]-	752.5231	6.62
	PE 37:5	[M-H]-	750.5074	6.03
	PE 38:1	[M-H]-	772.5856	7.82
	PE 38:2	[M-H]-	770.57	7.41
	PE 38:3	[M-H]-	768.5543	7.15
	PE 38:4	[M-H]-	766.5387	6.89
	PE 38:5	[M-H]-	764.5231	6.45
	PE 38:6	[M-H]-	762.5074	6.09
	PE 39:4	[M-H]-	780.5543	7.04
	PE 39:5	[M-H]-	778.5387	6.60
	PE 39:6	[M-H]-	776.5231	6.46
	PE 40:3	[M-H]-	796.5856	6.99
	PE 40:4	[M-H]-	794.57	7.35
	PE 40:5	[M-H]-	792.5543	7.05
	PE 40:6	[M-H]-	790.5387	6.81
	PE 40:7	[M-H]-	788.5231	6.32
	PE 40:8	[M-H]-	786.5074	5.91
	PE 42:8	[M-H]-	814.5387	6.43
	PE 42:9	[M-H]-	812.5231	6.00
PG	PG 33:0	[M-H]-	735.5176	6.08

	PG 34:1	[M-H]-	747.5176	5.88
	PG 34:2	[M-H]-	745.5019	5.47
	PG 36:0	[M-H]-	777.5646	6.90
	PG 36:1	[M-H]-	775.5489	6.47
	PG 36:2	[M-H]-	773.5333	6.04
	PG 36:3	[M-H]-	771.5176	5.41
	PG 36:4	[M-H]-	769.5019	5.46
	PG 38:4	[M-H]-	797.5333	6.07
PI	PI 34:1	[M-H]-	835.5337	5.72
	PI 34:2	[M-H]-	833.518	5.28
	PI 36:1	[M-H]-	863.5649	6.33
	PI 36:2	[M-H]-	861.5493	5.91
	PI 36:3	[M-H]-	859.5337	5.43
	PI 36:4	[M-H]-	857.518	5.27
	PI 38:3	[M-H]-	887.5649	6.12
	PI 38:4	[M-H]-	885.5493	5.89
	PI 38:5	[M-H]-	883.5337	5.41
	PI 40:6	[M-H]-	909.5493	5.83
pPE	pPE 32:0	[M-H]-	674.5124	7.04
	pPE 32:1	[M-H]-	672.4968	6.51
	pPE 34:0	[M-H]-	702.5438	7.25
	pPE 34:1	[M-H]-	700.5281	7.08
	pPE 34:2	[M-H]-	698.5124	6.64
	pPE 34:3	[M-H]-	696.4968	6.28
	pPE 36:0	[M-H]-	730.5751	8.04
	pPE 36:1	[M-H]-	728.5594	7.64
	pPE 36:2	[M-H]-	726.5438	7.24
	pPE 36:3	[M-H]-	724.5281	6.83
	pPE 36:4	[M-H]-	722.5124	6.59
	pPE 36:5	[M-H]-	720.4968	6.26
	pPE 38:1	[M-H]-	756.5907	8.14
	pPE 38:2	[M-H]-	754.5751	7.78
	pPE 38:3	[M-H]-	752.5594	7.42
	pPE 38:4	[M-H]-	750.5438	7.15
	pPE 38:5	[M-H]-	748.5281	6.66
	pPE 38:6	[M-H]-	746.5124	6.48
	pPE 40:3	[M-H]-	780.5907	7.70
	pPE 40:4	[M-H]-	778.5751	7.68
	pPE 40:5	[M-H]-	776.5594	7.24
	pPE 40:5	[M-H]-	776.5594	7.48
	pPE 40:6	[M-H]-	774.5438	7.10

	pPE 42:5	[M-H]-	804.5907	7.80
	pPE 42:6	[M-H]-	802.5751	7.68

[a] The mass accuracy was ±0.001 Da in positive mode and ±0.005 Da in negative mode, with overall mass error of less than 2 parts per million. CE: Cholesterol Esters; DAG: Diacylglycerol; LPC: Lysophosphatidylcholine; LPE: Lysophosphatidylethanolamine; MAG: Monoacylglycerol; PC: Phosphatidylcholine; pPC: Plasmenyl-Phosphatidylcholine; PE: Phosphatidylethanolamine; pPE: Plasmenyl-Phosphatidylethanolamine; PA: Phosphatidic Acid; SM: Sphingomyelin; TAG: Triacylglycerol; FFA: Free Fatty Acid; CerP: Ceramide-Phosphates; CL: Cardiolipin; PG: Phosphatidylglycerol; PI: Phosphatidylinositol

Supplement Table 2: Comparing z-score standardized mean and standard deviation (SD) of lipids in patients with and without stroke

	**Stroke, n=30**		**No Stroke, n=184**		
**Name**	**Mean**	**SD**	**Mean**	**SD**	**p value**
PC 38:4	0.62	0.88	-0.10	0.98	0.000189
pPE 38:2	-0.56	0.80	0.09	1.00	0.000759
PE 36:4	0.56	0.66	-0.09	1.02	0.000858
PC 34:0	0.55	0.99	-0.09	0.98	0.001061
PE 32:2	-0.55	1.27	0.09	0.92	0.001065
FFA 16:0	0.53	0.83	-0.09	1.00	0.001459
PE 38:1	-0.50	0.53	0.08	1.03	0.002832
pPE 40:3	-0.50	0.64	0.08	1.03	0.002853
pPC 20:0	-0.49	0.85	0.08	1.00	0.003861
DAG 38:4	0.47	1.10	-0.08	0.96	0.005528
pPE 36:4	0.44	0.91	-0.07	1.00	0.009651
pPE 38:1	-0.43	0.69	0.07	1.03	0.010048
PE 38:6	0.43	0.78	-0.07	1.02	0.011058
FFA 18:0	0.42	0.85	-0.07	1.01	0.012178
PC 40:7	0.42	0.82	-0.07	1.01	0.013356
PC 38:3	0.41	1.00	-0.07	0.99	0.014198
FFA 24:3	-0.41	1.44	0.07	0.90	0.015453
PC 40:3	0.41	0.89	-0.07	1.00	0.016098
PC 40:4	0.40	0.86	-0.06	1.01	0.018933
PA 34:0	0.39	1.06	-0.06	0.98	0.020787
PC 40:5	0.39	0.96	-0.06	0.99	0.021470
SM 34:2	0.38	0.91	-0.06	1.00	0.026220
CL 82:9	0.37	1.03	-0.06	0.99	0.027223
SM 36:2	0.37	0.94	-0.06	1.00	0.028680
DAG 38:5	0.37	1.04	-0.06	0.98	0.029836
DAG 38:3	0.36	1.09	-0.06	0.98	0.035297
PE 38:3	0.36	0.79	-0.06	1.02	0.035519
PC 32:0	0.35	1.24	-0.06	0.95	0.036238
PC 40:9	0.35	0.75	-0.06	1.03	0.037658
CL 74:3	-0.35	0.88	0.06	1.01	0.040648
PE 38:5	0.34	0.65	-0.06	1.04	0.041422
PE 38:2	-0.34	0.62	0.06	1.04	0.041509
PI 36:2	-0.34	1.17	0.06	0.96	0.041511
PC 24:0	-0.34	0.90	0.06	1.01	0.043630
PC 38:5	-0.33	1.06	0.05	0.98	0.048072
FFA 20:1	-0.33	0.78	0.05	1.02	0.049350
PA 34:2	-0.33	1.14	0.05	0.97	0.050875
pPE 38:4	0.33	0.72	-0.05	1.03	0.051422

PC 42:10	0.32	0.73	-0.05	1.03	0.056711
PI 36:4	0.32	0.80	-0.05	1.02	0.056921
PC 40:8	0.32	0.78	-0.05	1.02	0.060981
FFA 22:1	-0.31	0.70	0.05	1.03	0.067189
PE 38:4	0.31	0.83	-0.05	1.02	0.069792
LPC 20:1	-0.31	0.75	0.05	1.03	0.071491
PE 40:7	0.30	0.92	-0.05	1.01	0.076328
CerP 32:1	-0.30	0.81	0.05	1.02	0.077856
CerP 34:1	0.30	0.81	-0.05	1.02	0.077856
pPC 36:1	0.30	0.72	-0.05	1.03	0.078096
PC 32:3	-0.30	1.19	0.05	0.96	0.079023
SM 32:1	0.30	0.75	-0.05	1.03	0.079134
CL 82:13	0.30	0.84	-0.05	1.02	0.079744
PC 36:1	0.30	0.88	-0.05	1.01	0.079971
CE 22:4	0.30	0.88	-0.05	1.01	0.080204
SM 38:0	-0.30	1.16	0.05	0.97	0.080389
PE 34:4	-0.30	0.57	0.05	1.05	0.080822
PC 38:6	0.29	0.79	-0.05	1.02	0.082156
FFA 20:0	0.29	0.97	-0.05	1.00	0.082258
PE 40:6	0.29	0.95	-0.05	1.00	0.082925
PC 44:5	-0.29	1.16	0.05	0.97	0.083148
LPE 20:4	0.29	0.89	-0.05	1.01	0.090849
LPC 26:1	-0.29	1.13	0.05	0.97	0.091060
DAG 34:5	-0.28	2.30	0.05	0.55	0.093640
SM 40:4	0.28	0.88	-0.05	1.01	0.095762
PE 40:4	-0.28	0.84	0.05	1.02	0.096626
LPC 22:4	0.28	0.68	-0.05	1.04	0.097009
PC 38:8	0.28	0.67	-0.05	1.04	0.097850
CL 78:3	-0.27	0.89	0.04	1.01	0.106560
CL 78:5	-0.27	0.65	0.04	1.04	0.108180
TAG 54:0	0.27	0.80	-0.04	1.02	0.108850
LPC 16:0	0.27	0.91	-0.04	1.01	0.109390
DAG 32:3	-0.27	1.52	0.04	0.88	0.109740
FFA 24:0	0.27	0.65	-0.04	1.04	0.112430
pPE 38:5	0.26	0.57	-0.04	1.05	0.120240
pPE 40:4	0.26	1.06	-0.04	0.99	0.121330
PC 34:1	0.26	0.91	-0.04	1.01	0.123060
FFA 22:0	0.26	0.74	-0.04	1.03	0.127440
PC 40:6	0.26	0.87	-0.04	1.02	0.131780
TAG 56:1	-0.25	1.57	0.04	0.87	0.133690
PE 34:1	0.25	1.00	-0.04	1.00	0.139730
DAG 40:8	-0.25	1.04	0.04	0.99	0.142840
DAG 34:0	0.24	0.90	-0.04	1.01	0.152170

PC 42:11	0.24	0.80	-0.04	1.03	0.155730
pPE 42:6	0.24	0.90	-0.04	1.01	0.157890
CE 18:3	-0.24	0.87	0.04	1.02	0.160350
LPC 24:1	-0.24	0.84	0.04	1.02	0.162160
TAG 46:3	-0.23	1.12	0.04	0.98	0.169060
CE 20:4	0.23	0.90	-0.04	1.01	0.169090
SM 42:4	0.23	1.15	-0.04	0.97	0.169720
TAG 54:1	0.23	0.86	-0.04	1.02	0.171420
DAG 38:6	0.23	0.92	-0.04	1.01	0.173740
PG 38:4	-0.23	1.25	0.04	0.95	0.174580
DAG 38:1	-0.23	0.86	0.04	1.02	0.174670
LPC 20:5	0.23	0.98	-0.04	1.00	0.179220
PC 36:7	0.23	0.85	-0.04	1.02	0.181240
TAG 56:4	0.22	0.92	-0.04	1.01	0.186360
TAG 52:0	0.22	1.13	-0.04	0.98	0.189490
TAG 56:0	-0.22	1.35	0.04	0.93	0.190990
PC 36:4	0.22	1.23	-0.04	0.96	0.191680
CE 22:5	0.22	0.69	-0.04	1.04	0.194230
CL 74:7	0.22	0.74	-0.04	1.03	0.194700
CE 18:2	-0.22	1.04	0.04	0.99	0.201550
SM 42:2	0.22	0.95	-0.04	1.01	0.202930
PE 40:8	0.21	0.80	-0.03	1.03	0.215620
FFA 20:2	-0.21	0.96	0.03	1.00	0.222310
PE 36:1	0.21	1.02	-0.03	1.00	0.226050
PC 44:2	-0.21	0.84	0.03	1.02	0.226720
TAG 58:12	-0.20	1.02	0.03	1.00	0.229690
DAG 40:5	0.20	0.90	-0.03	1.01	0.232070
TAG 56:5	0.20	1.00	-0.03	1.00	0.233100
pPC 44:4	0.20	0.99	-0.03	1.00	0.234010
SM 34:0	-0.20	1.31	0.03	0.94	0.237060
PC 36:3	-0.20	0.91	0.03	1.01	0.237400
TAG 48:3	-0.20	1.20	0.03	0.96	0.237640
PC 32:2	-0.20	1.04	0.03	0.99	0.237980
PC 38:1	0.20	0.87	-0.03	1.02	0.238340
PE 34:2	0.20	0.92	-0.03	1.01	0.244900
PC 38:2	0.20	0.98	-0.03	1.00	0.246120
DAG 36:1	0.20	0.91	-0.03	1.01	0.248940
SM 38:1	-0.19	1.09	0.03	0.98	0.250580
DAG 30:0	-0.19	1.16	0.03	0.97	0.257550
PE 40:3	-0.19	1.01	0.03	1.00	0.261410
PC 26:0	-0.19	0.90	0.03	1.01	0.264550
FFA 18:2	-0.19	0.88	0.03	1.02	0.265390
LPC 14:0	-0.19	1.24	0.03	0.96	0.267330

FFA 24:1	0.19	0.84	-0.03	1.02	0.267390
TAG 48:2	-0.19	1.22	0.03	0.96	0.271980
PG 34:2	-0.18	0.92	0.03	1.01	0.278880
TAG 44:2	-0.18	1.31	0.03	0.94	0.279990
CE 16:0	0.18	0.80	-0.03	1.03	0.280840
PG 34:1	0.18	0.95	-0.03	1.01	0.290730
PC 34:2	-0.18	0.95	0.03	1.01	0.291470
DAG 34:4	0.18	0.78	-0.03	1.03	0.293280
LPC 18:0	0.18	1.02	-0.03	1.00	0.293930
DAG 40:7	-0.18	0.96	0.03	1.01	0.298580
TAG 56:6	0.18	1.05	-0.03	0.99	0.298890
pPE 42:5	0.17	1.03	-0.03	1.00	0.304910
pPE 38:3	0.17	0.73	-0.03	1.04	0.307330
PI 40:6	0.17	0.83	-0.03	1.02	0.316320
LPC 20:3	0.17	0.84	-0.03	1.02	0.319350
LPC 20:0	-0.17	1.08	0.03	0.99	0.325090
TAG 50:4	-0.16	1.10	0.03	0.98	0.336270
TAG 50:0	0.16	0.92	-0.03	1.01	0.339940
PI 38:3	-0.16	0.89	0.03	1.02	0.340250
LPE 18:2	-0.16	1.07	0.03	0.99	0.343530
TAG 56:3	0.16	0.74	-0.03	1.04	0.345530
CL 72:3	0.16	0.87	-0.03	1.02	0.349760
PC 40:1	-0.16	1.75	0.03	0.82	0.349860
PC 36:5	0.16	0.87	-0.03	1.02	0.351320
pPE 40:6	0.16	0.99	-0.03	1.00	0.351410
CE 20:5	-0.16	0.95	0.03	1.01	0.357500
PI 38:4	0.16	1.05	-0.03	0.99	0.360610
SM 34:1	0.15	1.03	-0.03	1.00	0.362210
FFA 22:2	-0.15	0.94	0.02	1.01	0.382340
pPE 36:2	-0.15	0.73	0.02	1.04	0.390470
LPE 20:5	-0.15	0.97	0.02	1.01	0.391350
PG 36:3	0.14	0.81	-0.02	1.03	0.394410
PC 44:4	-0.14	1.14	0.02	0.98	0.396580
TAG 50:3	-0.14	1.17	0.02	0.97	0.401790
PC 40:2	-0.14	1.15	0.02	0.97	0.401920
SM 30:1	-0.14	0.85	0.02	1.02	0.402900
PG 36:1	0.14	0.99	-0.02	1.00	0.404740
CE 20:1	0.14	0.99	-0.02	1.00	0.407480
TAG 52:1	0.14	0.79	-0.02	1.03	0.413420
PE 34:3	0.14	0.83	-0.02	1.03	0.413580
TAG 58:5	0.14	1.01	-0.02	1.00	0.418520
DAG 36:0	0.14	1.03	-0.02	1.00	0.418670
PI 34:2	-0.14	0.98	0.02	1.00	0.421260

TAG 54:2	0.14	0.88	-0.02	1.02	0.423590
TAG 62:14	0.13	0.61	-0.02	1.05	0.427690
PC 36:2	0.13	0.88	-0.02	1.02	0.440960
FFA 24:2	-0.13	0.91	0.02	1.02	0.443370
SM 42:1	0.13	0.91	-0.02	1.02	0.444680
DAG 32:1	-0.13	1.43	0.02	0.91	0.445780
LPE 18:3	-0.13	0.98	0.02	1.00	0.447100
SM 38:4	0.13	0.77	-0.02	1.03	0.447250
pPC 36:4	0.13	0.83	-0.02	1.03	0.449580
LPC 18:3	0.13	1.00	-0.02	1.00	0.450150
TAG 46:2	-0.13	1.25	0.02	0.96	0.451780
pPC 18:0	0.13	1.07	-0.02	0.99	0.452560
PA 34:1	-0.13	1.30	0.02	0.95	0.456510
LPE 22:6	-0.13	1.02	0.02	1.00	0.459130
TAG 48:1	-0.13	1.19	0.02	0.97	0.459260
PE 30:0	0.13	0.89	-0.02	1.02	0.459310
pPE 34:1	0.12	0.91	-0.02	1.01	0.465170
FFA 22:3	-0.12	0.84	0.02	1.02	0.466950
PC 42:2	-0.12	0.78	0.02	1.03	0.476590
PE 36:3	0.12	0.82	-0.02	1.03	0.477630
TAG 58:6	0.12	1.03	-0.02	1.00	0.478470
TAG 58:7	0.12	1.07	-0.02	0.99	0.485670
LPC 22:5	0.12	0.89	-0.02	1.02	0.486260
pPE 40:5	0.12	0.86	-0.02	1.02	0.486920
pPE 32:0	0.12	1.00	-0.02	1.00	0.494280
TAG 58:0	-0.12	0.97	0.02	1.01	0.496590
SM 40:1	0.11	1.03	-0.02	1.00	0.506710
PC 32:1	-0.11	1.18	0.02	0.97	0.508190
TAG 40:0	0.11	1.09	-0.02	0.99	0.510850
pPE 36:3	0.11	0.71	-0.02	1.04	0.517330
SM 38:5	0.11	0.90	-0.02	1.02	0.518360
PG 36:0	-0.11	0.88	0.02	1.02	0.519880
PE 32:0	-0.11	1.21	0.02	0.97	0.527860
TAG 46:1	-0.11	1.22	0.02	0.96	0.530400
TAG 50:5	-0.11	0.93	0.02	1.01	0.531520
LPE 22:4	0.11	1.32	-0.02	0.94	0.532670
PE 40:5	-0.10	0.88	0.02	1.02	0.537640
SM 44:1	-0.10	1.01	0.02	1.00	0.540080
TAG 58:10	0.10	0.89	-0.02	1.02	0.544990
PE 42:8	-0.10	1.03	0.02	1.00	0.554000
PC 36:6	0.10	0.86	-0.02	1.02	0.565830
LPE 24:0	0.10	0.89	-0.02	1.02	0.571050
MAG 18:1	0.10	1.32	-0.02	0.94	0.572380

MAG 18:2	-0.10	1.32	0.02	0.94	0.572380
pPE 36:1	-0.10	1.03	0.02	1.00	0.574110
LPC 22:0	-0.09	1.24	0.02	0.96	0.578680
PC 42:4	-0.09	0.99	0.02	1.00	0.580340
LPE 22:5	0.09	0.84	-0.02	1.02	0.581000
PC 34:3	0.09	0.77	-0.02	1.03	0.584560
FFA 20:4	-0.09	1.08	0.01	0.99	0.594520
LPC 16:1	0.09	0.98	-0.01	1.01	0.599960
PC 34:5	-0.09	0.74	0.01	1.04	0.601280
TAG 46:0	-0.09	1.26	0.01	0.95	0.604550
TAG 58:8	0.09	0.97	-0.01	1.01	0.608210
TAG 54:8	0.09	0.99	-0.01	1.00	0.608620
SM 28:0	-0.09	0.72	0.01	1.04	0.616160
CE 18:0	-0.08	0.87	0.01	1.02	0.623360
TAG 56:9	0.08	0.88	-0.01	1.02	0.625140
DAG 40:6	0.08	0.97	-0.01	1.01	0.629950
TAG 54:5	0.08	1.03	-0.01	1.00	0.630290
PE 36:5	0.08	0.83	-0.01	1.03	0.634880
TAG 58:11	0.08	0.78	-0.01	1.03	0.644510
TAG 58:9	0.08	0.94	-0.01	1.01	0.649040
PE 36:0	0.08	0.87	-0.01	1.02	0.651030
LPE 18:1	0.08	1.22	-0.01	0.96	0.652460
TAG 54:3	0.08	0.91	-0.01	1.02	0.654660
TAG 54:6	0.08	1.04	-0.01	1.00	0.655960
SM 32:0	-0.08	0.95	0.01	1.01	0.656060
DAG 36:6	-0.08	1.00	0.01	1.00	0.656660
TAG 60:10	0.07	0.98	-0.01	1.01	0.659460
TAG 58:4	0.07	0.79	-0.01	1.03	0.668630
TAG 56:7	0.07	1.03	-0.01	1.00	0.670200
TAG 60:3	0.07	0.81	-0.01	1.03	0.673460
DAG 32:0	0.07	1.03	-0.01	1.00	0.676090
DAG 34:2	-0.07	1.12	0.01	0.98	0.676160
DAG 34:3	-0.07	0.96	0.01	1.01	0.679510
TAG 48:0	-0.07	1.06	0.01	0.99	0.687590
PC 40:10	0.07	0.82	-0.01	1.03	0.691650
PC 38:7	0.07	1.06	-0.01	0.99	0.695000
DAG 40:0	-0.07	0.79	0.01	1.03	0.696310
LPC 20:2	-0.07	1.05	0.01	0.99	0.697440
PI 34:1	-0.07	0.83	0.01	1.03	0.697700
pPE 34:3	-0.06	0.86	0.01	1.02	0.709110
PE 32:1	0.06	1.01	-0.01	1.00	0.713490
SM 40:2	-0.06	0.96	0.01	1.01	0.714200
TAG 50:1	-0.06	0.88	0.01	1.02	0.718310

TAG 44:1	0.06	1.10	-0.01	0.99	0.719590
SM 42:5	0.06	0.83	-0.01	1.03	0.722410
pPE 34:0	0.06	0.87	-0.01	1.02	0.725290
DAG 36:2	0.06	0.82	-0.01	1.03	0.727150
TAG 54:4	0.06	0.97	-0.01	1.01	0.736080
PC 42:5	0.06	0.94	-0.01	1.01	0.738910
DAG 38:2	-0.06	0.81	0.01	1.03	0.745040
CL 74:1	0.05	0.79	-0.01	1.03	0.747410
TAG 54:7	0.05	1.10	-0.01	0.98	0.749900
CE 20:3	-0.05	1.23	0.01	0.96	0.756240
PC 28:0	-0.05	0.67	0.01	1.05	0.764960
CL 76:11	0.05	1.03	-0.01	1.00	0.765520
FFA 18:1	-0.05	0.78	0.01	1.03	0.765520
PG 36:4	0.05	0.85	-0.01	1.02	0.768500
PA 38:6	0.05	1.21	-0.01	0.97	0.771950
PE 42:9	-0.05	0.94	0.01	1.01	0.772260
TAG 50:2	-0.05	1.05	0.01	0.99	0.788700
pPE 38:6	0.05	0.80	-0.01	1.03	0.789090
LPE 16:0	0.05	1.02	-0.01	1.00	0.789500
DAG 28:1	0.05	0.89	-0.01	1.02	0.789740
PA 36:4	-0.04	1.06	0.01	0.99	0.793430
DAG 38:0	-0.04	0.86	0.01	1.02	0.802030
SM 44:2	0.04	0.74	-0.01	1.04	0.802420
DAG 38:7	0.04	0.84	-0.01	1.03	0.807860
PC 34:4	0.04	0.89	-0.01	1.02	0.807980
LPE 18:0	0.04	0.95	-0.01	1.01	0.808040
TAG 60:11	0.04	0.89	-0.01	1.02	0.808250
PG 36:2	0.04	0.80	-0.01	1.03	0.811270
TAG 52:2	0.04	1.04	-0.01	1.00	0.812660
TAG 56:2	0.04	0.80	-0.01	1.03	0.815880
CE 20:2	-0.04	0.91	0.01	1.02	0.820940
PC 42:9	0.04	1.14	-0.01	0.98	0.823540
CL 70:5	-0.04	0.85	0.01	1.02	0.831000
SM 32:2	-0.04	1.12	0.01	0.98	0.834680
CE 18:1	-0.03	0.96	0.01	1.01	0.844470
PC 30:0	0.03	1.06	-0.01	0.99	0.850630
TAG 60:8	-0.03	0.95	0.01	1.01	0.851220
DAG 30:1	0.03	0.89	-0.01	1.02	0.856090
TAG 42:1	-0.03	1.20	0.00	0.97	0.860070
TAG 62:3	-0.03	1.22	0.00	0.96	0.862410
CL 66:1	0.03	1.19	0.00	0.97	0.862500
pPE 32:1	-0.03	1.02	0.00	1.00	0.863070
TAG 58:1	-0.03	1.06	0.00	0.99	0.869410

PE 36:2	0.03	0.88	0.00	1.02	0.877450
PC 30:1	-0.02	0.89	0.00	1.02	0.883900
PC 42:7	0.02	1.03	0.00	1.00	0.887140
pPE 34:2	-0.02	0.77	0.00	1.03	0.891630
TAG 52:7	-0.02	0.81	0.00	1.03	0.893550
TAG 52:4	-0.02	1.04	0.00	1.00	0.895010
pPC 40:4	0.02	1.15	0.00	0.98	0.897610
LPC 24:0	0.02	0.82	0.00	1.03	0.898180
DAG 36:4	0.02	1.00	0.00	1.00	0.898870
CL 74:5	0.02	1.02	0.00	1.00	0.902990
SM 36:1	0.02	0.97	0.00	1.01	0.904510
TAG 60:12	-0.02	0.84	0.00	1.03	0.905840
LPE 20:3	-0.02	1.01	0.00	1.00	0.917840
TAG 52:3	0.02	1.02	0.00	1.00	0.920800
PI 38:5	0.02	1.02	0.00	1.00	0.923870
pPE 36:0	0.02	0.90	0.00	1.02	0.925860
DAG 36:5	-0.02	0.93	0.00	1.01	0.926630
CE 16:1	0.01	1.03	0.00	1.00	0.932090
PI 36:1	-0.01	0.63	0.00	1.05	0.943550
TAG 60:15	-0.01	0.87	0.00	1.02	0.951940
PI 36:3	-0.01	1.02	0.00	1.00	0.958150
pPE 36:5	-0.01	0.77	0.00	1.03	0.962390
CE 22:6	0.01	0.80	0.00	1.03	0.963430
TAG 56:8	0.01	0.95	0.00	1.01	0.963790
CE 16:2	0.01	1.03	0.00	1.00	0.963930
DAG 36:3	-0.01	0.93	0.00	1.01	0.964270
TAG 58:2	-0.01	0.77	0.00	1.03	0.964400
TAG 52:6	0.01	1.02	0.00	1.00	0.967950
DAG 32:2	-0.01	0.99	0.00	1.00	0.973880
CE 22:1	0.00	0.63	0.00	1.05	0.977860
TAG 62:12	0.00	0.75	0.00	1.04	0.984610
TAG 58:3	0.00	0.72	0.00	1.04	0.984900
TAG 52:5	0.00	0.99	0.00	1.00	0.985580
TAG 42:0	0.00	1.19	0.00	0.97	0.991920

CE: Cholesterol Esters; DAG: Diacylglycerol; LPC: Lysophosphatidylcholine; LPE: Lysophosphatidylethanolamine; MAG: Monoacylglycerol; PC: Phosphatidylcholine; pPC: Plasmenyl-Phosphatidylcholine; PE: Phosphatidylethanolamine; pPE: Plasmenyl-Phosphatidylethanolamine; PA: Phosphatidic Acid; SM: Sphingomyelin; TAG: Triacylglycerol; FFA: Free Fatty Acid; CerP: Ceramide-Phosphates; CL: Cardiolipin; PG: Phosphatidylglycerol; PI: Phosphatidylinositol
